# Modulation of Plasma and Milk Sphingolipids in Dairy Cows Fed High-Starch Diets

**DOI:** 10.3390/metabo11100711

**Published:** 2021-10-19

**Authors:** Jorge Eduardo Rico, Eveline C. Sandri, Andrea Celemín Sarmiento, Janie Lévesque, Ákos Kenéz, Daniel E. Rico

**Affiliations:** 1 Department of Animal and Avian Sciences, University of Maryland, College Park, MD 20742, USA; 2 CRSAD, Deschambault, QC G0A1S0, Canada; eveline.sandrin@coperdia.com.br (E.C.S.); acelemin06@unisalle.edu.co (A.C.S.); janie.levesque@crsad.qc.ca (J.L.); 3 Department of Infectious Diseases and Public Health, City University of Hong Kong, Hong Kong, China; akos.kenez@cityu.edu.hk

**Keywords:** high-starch diet, milk, ceramide, sphingomyelin

## Abstract

Bovine milk is a significant source of sphingolipids, dietary compounds that can exert anti-inflammatory actions, and which can modulate the host’s microbiome. Because sphingolipid synthesis can be modified by diet, we hypothesized that dietary conditions which reduced FFA availability may result in reduced sphingolipid synthesis. Twelve ruminally cannulated cows (120 ± 52 DIM; 35.5 ± 8.9 kg of milk/d; mean ± SD) were randomly assigned to treatment in a crossover design with 21-d periods. Treatments were (1) High starch (HS), (2) Control. The HS diet contained 29% starch, 24% NDF, and 2.8% fatty acids (FA), whereas the Control diet contained 20% starch, 31% NDF, and 2.3% FA. Plasma and milk samples were obtained on d 21 of each period and sphingolipids were quantified using targeted metabolomics. Univariate and multivariate analyses of generalized log-transformed and Pareto-scaled data included ANOVA (fixed effects of treatment) and discriminant analysis. The lipidomics analysis detected 71 sphingolipids across plasma and milk fat, including sphinganines (*n* = 3), dihydro-ceramides (*n* = 8), ceramides (Cer; *n* = 15), sphingomyelins (SM; *n* = 17), and glycosylated ceramides (*n* = 28). Followed by Cer, SM were the most abundant sphingolipids detected in milk and plasma, with a preponderance of 16:0-, 23:0-, and 24:0-carbon sidechains. Although no effects of HS diets were observed on plasma sphingolipids, we detected consistent reductions in the concentrations of several milk Cer (e.g., 22:0- and 24:0-Cer) and SM (17:0- and 23:0-SM) in response to HS. Discriminant analysis revealed distinct metabolite separation of HS and Control groups, with several Cer and SM being distinctively predictive of dietary treatment. We conclude that HS diets can reduce the secretion of milk Cer and SM, even in the absence of changes in circulating sphingolipids.

## 1. Introduction

Bovine milk is a nutrient-dense food that constitutes a major source of energy, high-quality protein, and vitamins and minerals in the human diet [1,2]. In addition to the classical nutritional benefits attributed to milk and milk products, the lipid fraction has sparked particular interest because it can be a source of health-promoting fatty acids, namely the conjugated linoleic acids and very-long chain omega-3 fatty acids [3,4,5]. More recently, bovine milk sphingolipids (e.g., sphingomyelins from the fat globular membrane) have received attention in basic research and clinical science. This is because of their ability to modulate microbial and host metabolism and physiology, including hypolipidemic effects on circulating and hepatic lipids, anti-inflammatory actions, the ability to modulate the microbiome to protect against gut dysbiosis, and even the potential to improve neurobehavioral development in human infants [6,7,8]. Furthermore, the consumption of sphingolipids of bovine origin appears to be advantageous when compared with other animal sources, such as egg yolk, as they are more effective in reducing the intestinal absorption of cholesterol in rodents [9].

Unlike the widely studied milk triglycerides, which comprise most milk lipids (~98%) [10], sphingolipids such as sphingosine, ceramide, glycosylated ceramides, and sphingomyelin, represent only a minor portion of the total (typically 0.5–1%) [11]. Moreover, our understanding of factors involved in the modulation of their metabolism remains limited. Recent research in dairy cows has shown that dietary manipulation can be used as a mean to influence sphingolipid synthesis, and particularly, that ceramide production can be promoted by increased availability of circulating fatty acids of endogenous or dietary origin (i.e., substrate-product relationships) [12,13,14]. Unfortunately, little is known about the extent to which dietary manipulation can be used to modulate milk sphingolipids.

Modern dairy cows are commonly fed higher energy diets to sustain elevated milk production. Relying substantially on grain feeding, feeding starch-rich diets carries an increased risk for the development of sub-acute ruminal acidosis (SARA) [15], which is due to accumulation of organic acids and/or insufficient rumen buffering [16]. Furthermore, high-grain diets are expected to increase the production of ruminal propionate to promote increased insulin secretion and reduce adipose tissue lipolysis and free fatty acid (FFA) concentrations, which may be important depending on lactation stage [17]. Indeed, we have recently reported that the induction of SARA with high-starch diets resulted in 52% higher plasma insulin and 32% lower plasma FFA [18]. Based on these observations we hypothesized that a high-starch diet will result in reduced FFA availability for sphingolipid synthesis, observed as reduced concentrations of plasma and milk sphingolipids. Consequently, our objective was to evaluate the effect of a high-starch diet on plasma and milk sphingolipid concentrations. 

## 2. Results

### 2.1. Animal Parameters and Metabolic Status

The time course of changes during the 3-wk feeding of a high-starch diet was previously reported by our group [18] Briefly, HS diet resulted in reduced daily ruminal pH, measured as increased total hours below pH 5.8. The HS diet increased total volatile FA yields in the rumen, as well as voluntary DMI, milk yield, milk protein concentration and yield, and circulating insulin, whereas circulating FFA tended to be lower, and overall milk fat yield was unchanged [18]. In agreement with our previous findings, we detected significantly higher DMI and milk yield in HS, relative to the Control (27.9 vs. 24.3 kg/d, and 34.4. vs. 30.3 kg/d, respectively) at d 21 of induction (*p* < 0.01). Similarly, plasma insulin, milk protein content, and milk protein yield were higher in HS, relative to control (3.42 vs. 3.35%, 1.15 vs. 0.99 kg/d, and 26 vs. 14.6 mg/dL, respectively: all *p* < 0.05). Furthermore, time below pH 5.8 in the reticulum was higher in HS relative to control (2.81 vs. 0.61 h/d; *p* < 0.0001).

### 2.2. Plasma and Milk Lipidome 

**Figure 1:**
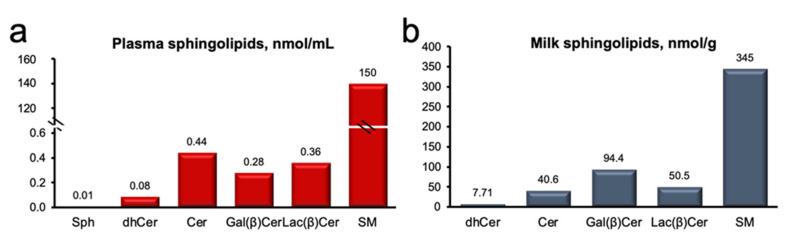
Sphingomyelin is the predominant sphingolipid of plasma and milk. (**a**) Plasma sphingolipids (nmol/mL) and (**b**) milk sphingolipids (nmol/g). Sphingosine (Sph), ceramide (Cer), galactosyl β-ceramide (Gal(β)Cer), lactosyl β-ceramide (Lac(β)Cer), and sphingolyelin (SM). Data are representative of plasma and milk collected from multiparous Holstein dairy cows (*n* = 12) at day 21 d. Lipidomics data were obtained using UPLC-MRM/MS with a UHPLC system and QTRAP mass spectrometry.

Our targeted lipidomics analysis detected 71 sphingolipids across plasma and milk fat, and included molecular species of sphinganine (SPH; 3), (Cer; 15), dihydro-ceramide (DH-Cer; 8), sphingomyelin (SM; 17), and glycosylated ceramides (galactosyl-, glucosyl-, and lactosyl-ceramides; GC; 28). We observed that SM was the predominant sphingolipid in both plasma and milk (Figure 1), representing 99% and 64% of total sphingolipids, respectively, and followed by Cer and GC. A detailed summary of treatment differences from ANOVA for plasma and milk sphingolipids are presented Tables S1 and S2 (Supplementary Materials). Overall, univariate analysis did not detect major changes in plasma sphingolipids (Table S1), except for 14:0- and 24:0-Cer, and 18:2 and 19:0 SM, all of which were reduced in HS, relative to the Control (*p* < 0.05). Conversely, significant reductions in several sphingolipids were detected in milk, with changes observed for 31 compounds across the DH-Cer, Cer, SM, and GC classes (*p* < 0.05; Table S2). 

**Figure 2:**
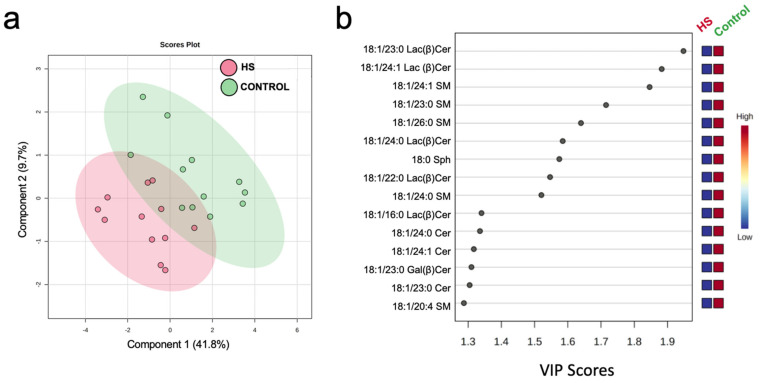
Discriminant analysis of milk sphingolipids of dairy cows following 3 weeks of high-starch (HS) feeding or Control. (**a**) Two-dimensional partial least squares discriminant (PLS-DA) score plot and (**b**) variable importance projection (VIP) scores analysis based on component 1 of the PLS-DA used to rank the relative contribution of metabolites to the variance between dietary treatments. Variance for component 1 is explained by changes in sphingosine (Sph), ceramide (Cer), galactosyl β-ceramide (Gal(β)Cer), and lactosyl β-ceramide (Lac(β)Cer), with odd and even side-chains. Normalized, pareto-scaled data are representative of milk collected from multiparous Holstein dairy cows (*n* = 12) at day 21 d. Lipidomics data were obtained using UPLC-MRM/MS with a UHPLC system and QTRAP mass spectrometry.

Multivariate analysis revealed a distinct effect of HS on milk sphingolipids, as evidenced by PLSDA scores plots and variable importance projection (VIP) scores in discriminant analysis (Figure 2). Variation in component 1 (41.8% of total variance; Figure 2a) was explained by unique changes in several molecular species of milk sphingosine (Sph), ceramide (Cer), galactosyl β-ceramide (Gal(β)Cer), and lactosyl β-ceramide (Lac(β)Cer), with long and very long and odd and even side-chains, that distinguished between HS and Control (Figure 2b). 

**Figure 3:**
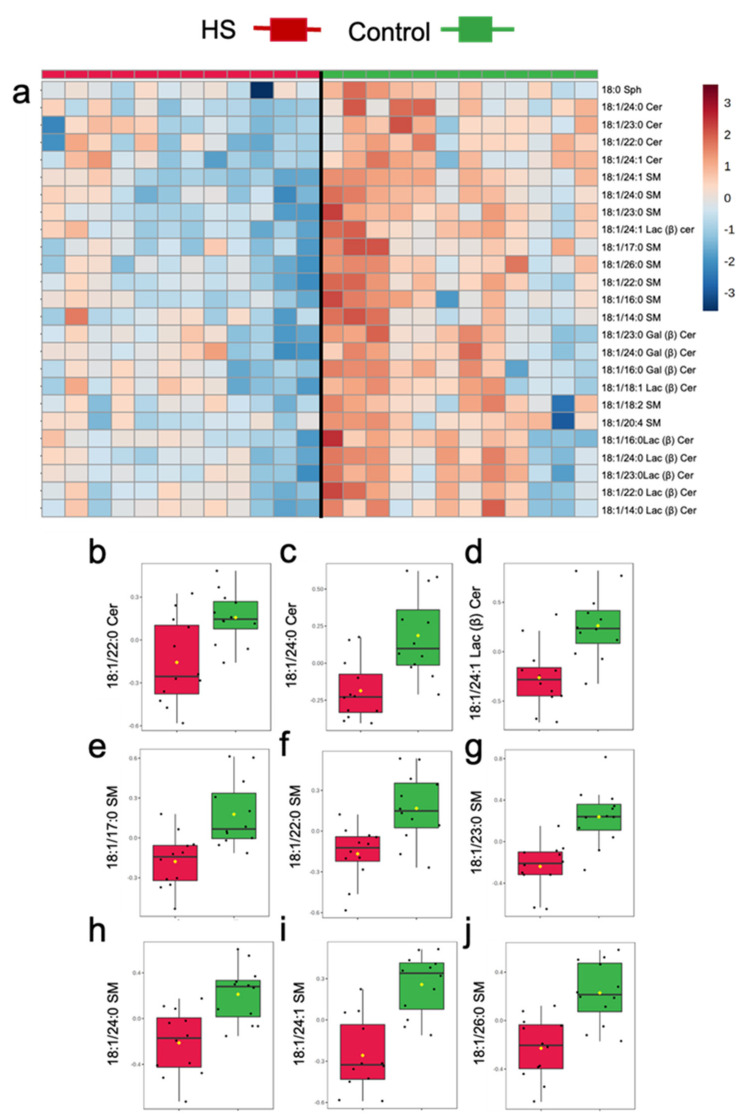
Differences in milk sphingolipids of dairy cows following 3 weeks of high starch-feeding (HS) feeding or Control. (**a**) Top 25 sphingolipid species that increased or decreased during HS feeding. (**b**–**j**) Treatment differences in milk ceramides (Cer), lactosyl β-ceramide (Lac(β)Cer), and sphingomyelin (SM) identified with the false discovery rate (FDR) method. Normalized, pareto-scaled concentration data, are representative of milk collected from multiparous Holstein dairy cows (*n* = 12) at day 21 d. For visualization purposes, the heat map represents log-transformed, pareto-scaled data to showcase concentrations in a color gradient as high (red) or low (blue). Panels represent significant treatments differences with FDR < 0.05. Lipidomics data were obtained using UPLC-MRM/MS with a UHPLC system and QTRAP mass spectrometry.

Similar to univariate analysis, multivariate statistics identified reductions in several milk sphingolipids in HS relative to the Control, particularly in several molecular species containing long acyl side chains (i.e., 22–26C; Figure 3). The general pattern of changes in milk sphingolipids is presented in Figure 3a, with an overall trend for reduced concentrations in milk sphingolipids during HS induction. Specifically, ANOVA detected significant reductions in the concentrations of odd and even chain very-long chain Cer, GC, and SM in HS, relative to the Control (FDR < 0.05; Figure 3b). On the contrary, no effects of HS induction were observed on plasma sphingolipids (all FDR > 0.25). 

## 3. Discussion

Milk fat is thought to be among the most complex food materials found in nature. Thanks to advances in analytical tools, especially high-resolution mass spectrometry, significant progress has been made in our ability to identify and quantify molecular species across several polar and neutral lipid classes, including phospholipids, sphingolipids, cholesterol, monoacylglycerol, diacylglycerol, triacylglycerol, free fatty acids, cholesteryl esters, and hydrocarbons [10,11]. Our metabolomics approach allowed us to characterize and quantify a wide range of milk sphingolipids, comprising most of the previously reported lipid classes [10], except for gangliosides and cerebrosides. Furthermore, we confirmed the predominance of SM in both plasma and milk lipids, in agreement with previous reports [10,11,19]. Relevant to our research question, SM is a key sphingolipid class recently reported to exert health-promoting effects [6,7,8], and thus constituting an important target for the evaluation of strategies that influence the secretion of bioactive compounds in bovine milk.

The impact of dietary factors on sphingolipid content of bovine milk has not been extensively studied. In an evaluation of factors influencing milk sphingomyelin content, Graves et al. [20] reported not detecting significant changes in sphingomyelin content of milk fat in response to diet, despite feeding dairy cows with a relatively high amount of soybean oil (i.e., 4% of dietary dry matter). Although numerical increases in sphingomyelin content of milk fat were evident, it is possible that this study was restricted by a small sample size (*n* = 6) and limited statistical power to detect potential differences. In contrast, and in agreement with our hypothesis, feeding a high-starch diet appeared to influence sphingolipid metabolism in our study. Although we cannot be certain about the causes for the observed reductions in milk Cer and SM, it is conceivable that an increased supply of rapidly fermentable carbohydrates—mainly in the form of starch—could reduce the availability of adipose tissue-derived FFA for de novo sphingolipid synthesis via increased pancreatic insulin secretion. Indeed, we and others have previously observed increased insulin and reduced FFA concentrations in plasma of cows fed high-starch diets [18,21], and a higher incidence of laminitis associated with reduced circulating sphingomyelin concentrations in fattening Holstein bulls fed a high-starch and high-protein diet [22]. In addition to limitations in substrate availability for Cer and SM synthesis, we posit that high-starch diets could potentially induce a proinflammatory status in dairy cows [23,24] that may in turn induce SM degradation. This mechanism appears to include the activation of acid and neutral sphingomyelinases by tumor necrosis factor α (TNFα) [25,26,27]. Because high-concentrate feeding can result in increased plasma concentrations of lipopolysaccharide binding protein (LBP) [18], a proxy for increased LPS entry to the bloodstream (i.e., endotoxemia), we speculate that the induction of the proinflammatory state that promotes SM degradation may be the result of LPS activation of toll-like receptor-4 signaling and TNFα [28]. In line with this, ruminal acidosis induced by high-grain diets increases gut permeability, leading to endotoxemia and inflammation [24,29,30]. Similarly, we observed reduced pH in the rumen [18] and in the reticulum in cows fed the high starch diet, as well as increased levels of LBP. Under this scenario, increased insulin concentrations may be explained both by the increased ruminal propionate concentrations [31] and by direct induction of insulin release in pancreatic B-cells via cytokine release (e.g., interleukin 1-β) from white blood cells [32]. The extent to which these mechanisms may account for the observed reductions in sphingolipid concentrations remains to be investigated. Furthermore, the reasons for the small effect of diet on the concentrations of plasma sphingolipids relative to those observed in milk are not clear, although we speculate that changes in FFA availability may impact milk lipids to a higher extent because available FFA are readily directed to and utilized by the mammary gland during lactation [33]. 

Although some of the diet-induced changes observed in plasma sphingolipids were also evidenced in milk, it is not clear whether changes observed in milk sphingolipids can be explained by those detected in plasma. It is conceivable that sphingolipids of hepatic origin—synthesized from FFA and readily available in circulation—could be taken by the mammary gland and incorporated into the sphingolipids of the milk globular membrane. In support of this possibility, changes in 24:0 Cer, a major ceramide of plasma [14], were directionally consistent in plasma and milk fat. Nevertheless, most changes in sphingolipids in response to treatment were observed in milk fat, and only a fraction in plasma, which may indicate that sphingolipid metabolism in the mammary gland could be largely independent of sphingolipid supply for peripheral circulation. Furthermore, we consider that our data support the possibility FFA may be the major factor responsible for the reductions observed in milk sphingolipids, and that the contribution from plasma sphingolipids may be small and limited only to the uptake of smaller sphingolipids—such as sphingosine and sphinganine—, that may serve as primers for the synthesis of ceramides and sphingomyelins. This possibility would be in keeping with the reported breakdown of complex lipids during intestinal digestion by sphingomyelinases and ceramidases and the subsequent absorption of FFA and sphingosine backbones [34]. Future investigations should aim to elucidate the extent to which plasma lipids are used by the mammary gland, as well as the contribution of this tissue to sphingolipid metabolism and secretion in milk fat. 

## 4. Materials and Methods

### 4.1. Experimental Design and Treatments

Twelve ruminally cannulated Holstein cows (120 ± 52 DIM; 35.5 ± 8.9 kg of milk/d; mean ± SD) from the dairy herd of the Centre de Recherche en Sciences Animals de Deschambault (CRSAD, Deschambault, QC, Canada) were used in this study. The experiment was conducted from December 2016 to March 2017, as previously described [18]. Cows were housed individually in tie stalls and had continuous access to water. Animals were randomly assigned to dietary treatment in a crossover arrangement to receive either a high-starch or a control diet. Experimental periods were 21-d in length. Dietary treatments are presented in Table 1 and were as follows: (1) the high-starch diet (**HS**; 29.4% starch, 24.0% NDF, and 2.8% FA); (2) low-starch diet (**Control**; 20.0% starch, 31.0% NDF, and 2.6% FA). 

### 4.2. Management, Feeding and Blood and Milk Sampling

Cows were fed once daily (1000 h) at 110% of expected intake with total mixed ration (TMR). Diet composition was adjusted weekly based on the variation in DM content of forages. The diets were composed of corn silage, alfalfa haylage, soybean meal, corn gluten, ground corn and a commercial vitamin/mineral mix (Table 1). Dry matter intake (DMI) was determined as the difference between DM offered and refused. Cows were milked twice daily at 730 h and 1630 h and milk yield was recorded as previously described [18]. Milk was sampled on each of 2 daily milkings. One subsample was stored at 4 °C with the preservative Bronopol (2-bromo-2-nitropropane-1,3-dio) until analyzed for fat, protein, lactose, and β-hydroxybutyrate (BHBA; Lactanet, Sainte-Anne-de-Bellevue, QC, Canada), and a second aliquot was used for cream separation. Milk cream was obtained by centrifugation at 12,300 rpm for 30 min, at 3 °C, and stored at −20 °C without preservative for metabolomics analyses. Blood was obtained by coccygeal venipuncture using a 10 mL Vacutainer tube containing ethylenediamine tetraacetic acid (EDTA). Blood samples were immediately placed on iced and centrifuged at 1800× *g* for 15 min at 4 °C. 

### 4.3. Experimental Analyses

#### 4.3.1. Ruminal and Reticular pH

pH was monitored using indwelling probes (eCow Devon Ltd., Devon, UK) placed in the ventral sac of the rumen and the reticulum through the rumen cannula, starting at -30 min relative to feeding time (at approximately 09h30). Ruminal boluses were attached to weights to prevent migration into the reticulum, whereas reticular boluses were placed directly in the reticulum. pH was recorded every 5 min during 24 h on days 0, 3, 7, 14, and 21 of each period. The calibration of the boluses was verified daily (pH 4.0 and 7.0 at 39 °C) before inserting them in the cows and at the end of the measurement period. Data were discarded when outside of ±0.1 pH units from either calibration point at the time of removal from the cow. 

#### 4.3.2. Lipidomics

Lipidomics analyses of plasma and milk fat cakes were performed at The Metabolomics Innovation Center (TMIC; UVic Node at The University of Victoria, Genome BC Proteomics Centre, Victoria, BC, Canada). First, 50 mg of each fat cake sample were precisely weighed into a 2-mL homogenizing tube. Then, 1 mL of methanol-chloroform (3:2) containing 1 mg/mL BHT and two 4-mm metal beads were added. Samples were homogenized on a MM 400 mill mixer at a shaking frequency of 30 Hz for 1 min, three times, followed by sonication for 2 min in an ice-water bath. After centrifugation in an Eppendorf 5420R centrifuge for 15 min at 21,000× *g* and 10 °C, the clear supernatant was collected. Next, 100 µL of plasma were added with 1 mL of methanol-chloroform (2:1) containing 1 mg/mL BHT and two 4-mm metal beads were added. Samples were homogenized on a MM 400 mill mixer at a shaking frequency of 30 Hz for 1 min, three times, followed by sonication for 2 min in an ice-water bath. The clear supernatant was transferred to an LC injection vial and was dried down under a gentle nitrogen gas flow at 30 °C. The residue was reconstituted in 250 µL of methanol-chloroform (3:2). A mixed standard stock solution (60 standards) was prepared for targeted analyses that included sphinganines, glucosylsphingosine, galactosylsphingosine, ceramides, ceramide phosphate, glucosylceramide, galactosylceramides, lactosylceramides, dihydrosphingomyelin, lyso-sphingomyelin, and dihydro lyso-sphingomyelin (Avantis Polar Lipids, Alabaster, AL, USA; Cayman chemical). The standard stock solution was prepared at a concentration of 20 nmol/mL (S1) for each analyte in methanol-chloroform (3:2) containing 1 mg/mL BHT. This solution was serially diluted in a ratio of 1:4 (*v/v*) with the same solvent to have standard solutions of S2 to S10. 6-μL aliquots of the standard solutions and the sample solutions were injected onto a UPLC column (C8, 2.1 × 50 mm, 1.7 um) to run UPLC-MRM/MS on an Agilent 1290 UHPLC system coupled to a 4000 QTRAP mass spectrometer, which was operated in the multiple-reaction monitoring (MRM) mode with positive-ion detection. The mobile phase was 0.01% formic acid in water and acetonitrile-isopropanol (2:1) for binary-solvent gradient elution (40% to 100% organic solvent in 12 min followed by 4-min column equilibration, at 400 µL/min and 60 °C). The ion transitions for MRM detection of each sphingolipid were optimized by direction infusion of the sphingolipid standard solutions to have two ion transitions per compound. UPLC-MRM/MS data were recorded using the Sciex Analyst software and were processed using the Sciex MultiQuant software. Linear-regression calibration curves of individual sphingolipids were constructed with external calibration, and the concentrations of sphingolipids detected in each sample were calculated from the calibration curves with the measured peak areas. Representative extracted ion chromatograms (XIC) with ion transitions for plasma and milk sphingolipids using UPLC-MRM/MS, are shown in Figures S1 and S2, respectively (Supplementary Materials). 

### 4.4. Statistical Analyses

#### 4.4.1. Univariate Data

Data were analyzed using the MIXED procedure of SAS (version 9.3, SAS Institute, Cary, NC, USA). The model included cow and period as random effects, and the fixed effect of treatment. The Kenward–Rogers method was used for adjustment of denominator degrees of freedom. Data were considered as outliers and removed from analysis when studentized residuals were >3.0 or <−3.0. Data were log transformed if distribution of residuals was non-constant and back-transformed values are reported. Significance and tendencies of main effects were declared at *p* ≤ 0.05 and 0.05 < *p* ≤ 0.15, respectively. Results are expressed as least squares means and their standard errors.

#### 4.4.2. Multivariate Data

We analyzed lipidomic data using the web server MetaboAnalyst 5.0 (www.metaboanalyst.ca; accessed on 15–17 January 2021 [35]). Non-filtered data were normalized by the sum method, generalized log-transformed, and Pareto-scaled. Multivariate analysis of data included partial least squares discriminant analysis (PLS-DA), ANOVA, and Pearson’s correlation coefficient procedures. Significance was declared at *p* ≤ 0.05 and false discovery rate (FDR) at < 0.05. For visualization purposes, heat maps were generated using generalized log-transformed, normalized and pareto-scaled data to showcase the magnitude of fold-change in a color gradient for increased (red) or decreased (blue) relative abundance. 

## 5. Conclusions

We conclude that high-starch diets can influence the composition of plasma and milk sphingolipids. These changes are associated with classical observations under these types of diets, such as reduced ruminal pH, increased ruminal propionate concentrations, as well as reduced lipid mobilization via insulin action. Indeed, we posit that reduced lipid mobilization under these conditions may largely explain the observed reductions in the concentrations of several milk sphingolipids. The increase in diet fermentability (i.e., high-starch dies), is commonly associated with ruminal acidosis, a condition for which the milk biomarkers identified herein could be of interest. Future studies should evaluate the usefulness of these biomarkers in terms of their prognostic and diagnostic value for acidosis and other metabolic disorders and validate them using independent and larger animal cohorts.

Our data support a direct relationship between diet, intermediary metabolism and milk sphingolipids, thus providing a framework for the future evaluation of nutritional strategies to modulate the synthesis and secretion of polar lipids in milk.

## Figures and Tables

**Table 1:** Ingredient and chemical composition of experimental diets ^1^: high starch (HS) and low-starch diet (Control).

Item	HS	Control
Ingredients, % of DM		
Corn silage	34.6	44.7
Alfalfa silage	22.2	24.5
Ground corn	21.7	4.67
Grass hay	5.10	10.5
Corn gluten meal	6.41	7.86
Soybean meal	7.31	5.08
Limestone	0.62	0.61
Mineral and vitamins mix ^2^	2.06	2.04
Chemical composition, % of DM		
OM	93.4	92.7
CP	16.1	17.2
NDF	24.0	31.0
Total FA	2.80	2.57
Starch	29.4	20.0

DM = dry matter; OM = organic matter; CP = crude protein; NDF = neutral detergent fiber; ADF = acid detergent fiber; FA = fatty acids; ^1^ *n* = 3 per diet; ^2^ contained (DM basis): 18.0% Ca, 5.0% P, 9.5% Na, 6.0% Mg, 45 mg/kg I, 3620 mg/kg Fe, 600 mg/kg Cu, 2000 mg/kg Mn, 3000 mg/kg Zn, 20 mg/kg Co, 480 mg/kg F, 25 mg/kg Se, 300,000 IU/kg vitamin A—retinol acetate, 100,000 IU/kg vitamin D—D3 cholecalciferol, and 1500 IU/kg vitamin E—DL-alpha tocopherol acetate (La coop fédérée, QC, Canada).

## Data Availability

Data is contained within the article or Supplementary Materials.

## Supplementary Materials

The following are available online at www.mdpi.com/article/10.3390/metabo11100711/s1, Table S1: Sphingolipid concentrations in plasma following 3 weeks of high-starch (HS) feeding or Control.; Table S2: Sphingolipid concentrations in milk fat following 3 weeks of high-starch (HS) feeding or Control. Figure S1: Representative extracted ion chromatogram (XIC) with ion transitions for plasma sphingolipids using UPLC-MRM/MS. Figure S2: Representative extracted ion chromatogram (XIC) with ion transitions for milk fat cake sphingolipids using UPLC-MRM/MS.
