# Supplementary material for: Modulation of Plasma and Milk Sphingolipids in Dairy Cows Fed High-Starch Diets
Source: Metabolites. 2021 Oct 19;11(10):711. doi: 10.3390/metabo11100711 (PMC8540507; doi:10.3390/metabo11100711)
Supplement: Supplementary file 1 [file metabolites-11-00711-s001.zip › metabolites-1400160-supplementary.pdf]

Supplementary Materials

Table S1. Sphingolipid concentrations in plasma following 3 weeks of high-starch (HS) feeding or Control. Sphingosine (Sph), dihydro ceramide (dhCer), ceramide (Cer), galactosyl β-ceramide (Gal(β)Cer), lactosyl β-ceramide (Lac(β)Cer), and sphingomyelin (SM). Data are presented as least square means and standard error of blood collected from multiparous Holstein dairy cows (n = 12) at day 21 d. Lipidomics data were obtained using UPLC-MRM/MS with a UHPLC system and QTRAP mass spectrometry.

Concentration, nmol/mL	SARA	Control	SEM	*P*-value
SPH(d16:0)	0.0024	0.0037	0.0006	0.30
SPH (d18:0)	0.0031	0.0023	0.0004	0.33
SPH(d20:0)	0.0023	0.0020	0.0002	-
dhCer(d18:0/22:0)	0.0173	0.0220	0.0023	0.16
dhCer(d18:0/24:0)	0.0630	0.0668	0.0054	0.63
Cer(d18:1/14:0)	0.0123	0.0193	0.0014	0.05
Cer(d18:1/16:0)	0.1405	0.1726	0.0214	0.31
Cer (d18:1/18:0)	0.0072	0.0067	0.0009	0.79
Cer(d18:1/22:0)	0.0114	0.0104	0.0019	0.69
Cer(d18:1/23:0)	0.0216	0.0214	0.0035	0.94
Cer(d18:1/24:0)	0.0335	0.0454	0.0061	0.05
Cer(d18:1/24:1(15Z))	0.0201	0.0214	0.0021	0.48
SM(d18:1/12:0)	0.3797	0.3745	0.0474	0.87
SM(d18:1/14:0)	4.40	4.49	0.3986	0.83
SM(d18:1/16:0)	31.6	33.7	2.1454	0.35
SM(d18:1/16:1)	6.33	6.51	0.3401	0.75
SM(d18:1/17:0)	32.6	31.6	1.3071	0.50
SM(d18:1/18:0)	3.33	3.39	0.1830	0.66
SM(d18:1/18:1)	5.88	6.21	0.4695	0.13
SM(d18:1/18:2)	0.87	1.06	0.0959	0.00
SM(d18:1/19:0)	1.47	1.72	0.1251	0.02
SM(d18:1/20:0)	0.72	0.73	0.0753	0.88
SM(d18:1/20:1)	0.78	0.85	0.0489	0.11
SM(d18:1/20:4)	0.08	0.10	0.0069	0.11
SM(d18:1/22:0)	4.41	4.68	0.3606	0.43
SM(d18:1/23:0)	25.1	28.7	2.2655	0.18
SM(d18:1/24:0)	17.8	18.9	1.1853	0.41
SM(d18:1/24:1)	9.40	10.96	0.6981	0.10
SM(d18:1/26:0)	0.73	0.79	0.0935	0.24
Glc(β)Cer(d18:1/18:0)	0.0020	0.0024	0.0004	0.36
Gal(β)Cer(d18:1/22:0)	0.0644	0.0696	0.0097	0.59
Gal(β)Cer(d18:1/23:0)	0.0727	0.0768	0.0125	0.66
Gal(β)Cer(d18:1/24:0)	0.1193	0.1515	0.0185	0.31
Lac(β)Cer(d18:1/14:0)	0.0922	0.0692	0.0108	0.20
Lac(β)Cer(d18:1/16:0)	0.1388	0.1489	0.0080	0.35

Lac(β)Cer(d18:1/17:0)	0.0178	0.0137	0.0037	0.14
Lac(β)Cer(d18:1/18:0)	0.0141	0.0192	0.0027	0.20
Lac(β)Cer(d18:1/18:1)	0.0113	0.0149	0.0030	0.48
Lac(β)Cer(d18:1/22:0)	0.0041	0.0040	0.0006	0.75
Lac(β)Cer(d18:1/23:0)	0.0057	0.0075	0.0008	-
Lac(β)Cer(d18:1/24:0)	0.0359	0.0343	0.0080	0.87
Lac(β)Cer(d18:1/24:1)	0.0392	0.0534	0.0107	0.17

Table S2. Sphingolipid concentrations in milk fat following 3 weeks of high-starch (HS) feeding or Control. Sphingosine (Sph), dihydro ceramide (dhCer), ceramide (Cer), galactosyl β-ceramide (Gal(β)Cer), lactosyl β-ceramide (Lac(β)Cer), and sphingomyelin (SM). Data are presented as least square means and standard error of milk collected from multiparous Holstein dairy cows (n = 12) at day 21 d. Lipidomics data were obtained using UPLC-MRM/MS with a UHPLC system and QTRAP mass spectrometry.

Concentration, nmol/g	SARA	Control	SEM	*P*-value
dhCer(d18:0/12:0)	0.53	0.60	0.09	0.372
dhCer(d18:0/14:0)	0.50	0.56	0.05	0.068
dhCer(d18:0/16:0)	0.57	0.63	0.04	0.064
dhCer(d18:0/17:0)	0.24	0.26	0.02	0.697
dhCer(d18:0/20:0)	2.55	2.70	0.29	0.437
dhCer(d18:0/22:0)	2.25	2.77	0.22	0.007
dhCer(d18:0/24:0)	0.57	0.68	0.05	0.026
Cer (d18:1/12:0)	0.02	0.02	0.00	0.793
Cer(d18:1/14:0)	0.38	0.44	0.04	0.219
Cer(d18:1/16:0)	21.60	23.97	1.86	0.057
Cer(d18:1/17:0)	0.12	0.14	0.01	0.092
Cer (d18:1/18:0)	1.67	1.83	0.14	0.146
Cer(d18:1/19:0)	0.30	0.28	0.02	0.662
Cer(d18:1/20:0)	0.12	0.12	0.01	0.541
Cer(d18:1/21:0)	0.23	0.25	0.02	0.237
Cer(d18:1/22:0)	2.70	3.29	0.13	0.001
Cer(d18:1/23:0)	5.51	7.47	0.52	0.001
Cer(d18:1/24:0)	3.59	4.71	0.19	0.003
Cer(d18:1/24:1(15Z))	1.04	1.40	0.10	<0.0001
Cer(d18:1/26:0)	0.03	0.04	0.00	0.428
SM(d18:1/12:0)	1.00	1.18	0.10	0.163
SM(d18:1/14:0)	17.0	21.0	1.79	0.047
SM(d18:1/16:0)	61.2	79.5	5.19	0.002
SM(d18:1/16:1)	2.63	3.25	0.37	0.159
SM(d18:1/17:0)	32.1	40.9	1.57	0.001
SM(d18:1/18:0)	6.16	6.30	0.20	0.473
SM(d18:1/18:1)	1.70	1.88	0.09	0.024

SM(d18:1/18:2)	0.16	0.20	0.01	0.008
SM(d18:1/19:0)	1.88	2.01	0.11	0.221
SM(d18:1/20:0)	11.63	13.03	0.91	0.039
SM(d18:1/20:1)	0.68	0.69	0.04	0.824
SM(d18:1/20:4)	0.25	0.40	0.04	0.004
SM(d18:1/22:0)	26.8	33.4	1.31	0.0001
SM(d18:1/23:0)	83.2	123.0	6.27	<0.0001
SM(d18:1/24:0)	38.1	52.7	2.73	<0.0001
SM(d18:1/24:1)	9.34	14.12	0.66	<0.0001
SM(d18:1/26:0)	0.71	1.02	0.05	0.000
Gal(β)Cer(d18:1/12:0)	0.03	0.04	0.00	0.265
Gal(β)Cer(d18:1/14:0)	0.40	0.50	0.05	0.097
Gal(β)Cer(d18:1/16:0)	8.8	12.0	0.92	0.004
Glc(β)Cer(d18:1/18:0)	0.53	0.57	0.05	0.337
Glc(β)Cer(d18:1/18:1(9Z))	0.24	0.24	0.03	0.810
Glc(β)Cer(d18:1/19:0)	0.05	0.05	0.01	0.889
Glc(β)Cer(d18:1/20:0)	0.28	0.29	0.03	0.711
Glc(β)Cer(d18:1/21:0)	0.31	0.36	0.03	0.213
Gal(β)Cer(d18:1/22:0)	28.0	36.0	2.73	0.022
Gal(β)Cer(d18:1/23:0)	26.4	38.5	3.12	0.005
Gal(β)Cer(d18:1/24:0)	13.6	17.8	1.49	0.013
Gal(β)Cer(d18:1/24:1(15Z))	1.44	1.66	0.14	0.321
Gal(β)Cer(d18:1/25:0)	0.24	0.40	0.06	0.095
Lac(β)Cer(d18:1/14:0)	0.81	1.24	0.14	0.020
Lac(β)Cer(d18:1/16:0)	4.69	8.66	1.20	0.033
Lac(β)Cer(d18:1/17:0)	0.60	0.96	0.13	0.027
Lac(β)Cer(d18:1/18:0)	1.27	1.41	0.15	0.190
Lac(β)Cer(d18:1/18:1)	1.61	2.14	0.19	0.005
Lac(β)Cer(d18:1/19:0)	0.09	0.11	0.01	-
Lac(β)Cer(d18:1/20:0)	1.93	2.28	0.14	0.114
Lac(β)Cer(d18:1/21:0)	0.76	0.97	0.08	0.043
Lac(β)Cer(d18:1/22:0)	10.2	19.1	2.31	0.016
Lac(β)Cer(d18:1/23:0)	7.02	16.05	1.74	0.002
Lac(β)Cer(d18:1/24:0)	6.06	9.79	0.84	0.001
Lac(β)Cer(d18:1/24:1)	0.94	1.56	0.12	<.0001
Lac(β)Cer(d18:1/25:0)	0.24	0.36	0.04	0.015
Lac(β)Cer(d18:1/26:0)	0.07	0.11	0.02	0.116

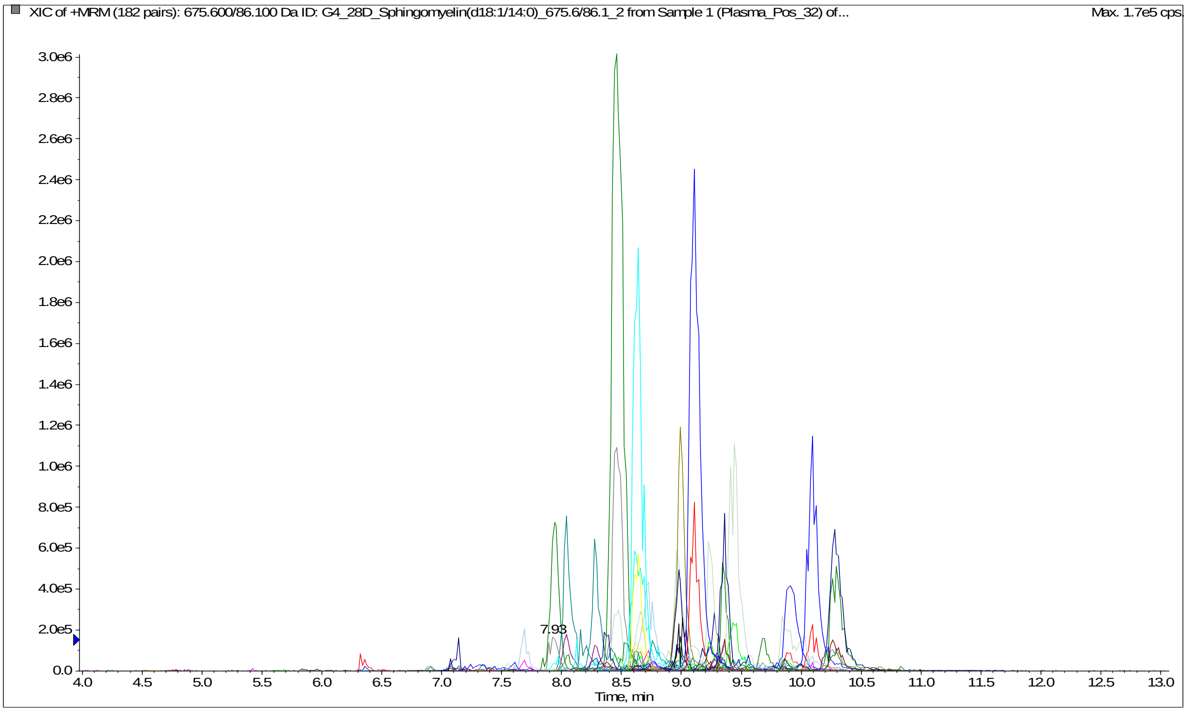

Figure S1. Representative extracted ion chromatogram (XIC) with ion transitions for plasma sphingolipids using UPLC-MRM/MS. Samples analyzed correspond to plasma of cows following 3 weeks of high-starch (HS) feeding or Control. Lipidomics data were obtained using UPLC-MRM/MS with a UHPLC system and QTRAP mass spectrometry operated in positive-ion mode.

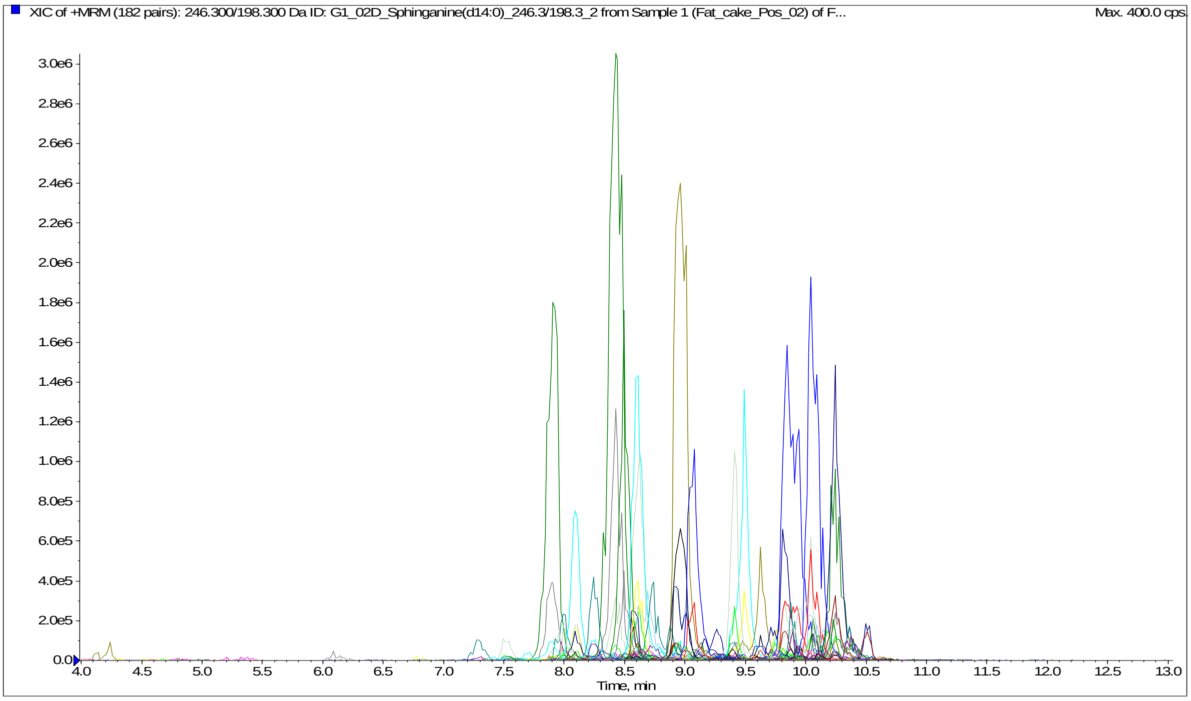

Figure S2. Representative extracted ion chromatogram (XIC) with ion transitions for milk fat cake sphingolipids using UPLC-MRM/MS. Samples analyzed correspond to milk of cows following 3 weeks of high-starch (HS) feeding or Control. Lipidomics data were obtained using UPLC-MRM/MS with a UHPLC system and QTRAP mass spectrometry operated in positive-ion mode.
